# The Plant Growth-Promoting Bacterium *Bacillus cereus* LpBc-47 Can Alleviate the Damage of Saline–Alkali Stress to *Lilium pumilum*

**DOI:** 10.3390/microorganisms13061248

**Published:** 2025-05-28

**Authors:** Miaoxin Shi, Lingshu Zhang, Hao Sun, Shangwei Ji, Huitao Cui, Wenhao Wan, Xingyu Liu, Ao Tian, Wei Yang, Xinran Wang, Fengshan Yang, Shumei Jin

**Affiliations:** 1 Key Laboratory of Saline-Alkali Vegetation Ecology Restoration of the Ministry of Education, College of Life Sciences, Northeast Forestry University, Harbin 150028, China; shimiaoxin0224@163.com (M.S.); 15942937576@163.com (L.Z.); hao870882@163.com (H.S.); jswx2022@163.com (S.J.); meow0198@163.com (H.C.); wwh13589976069@163.com (W.W.); nku1dsa@163.com (X.L.); ta15845265201@163.com (A.T.); 2 Heilongjiang Agricultural Technology Extension Station, Harbin 150090, China; yxwyyy@126.com; 3 Chinese and Binlingual Study (CBS), The Hong Kong Polytechnic University, Hong Kong SAR 999077, China; 22102991d@connect.polyu.hk; 4 Engineering Research Center of Agricultural Microbiology Technology, Ministry of Education, Heilongjiang University, Harbin 150080, China; 5 Heilongjiang Provincial Key Laboratory of Ecological Restoration and Resource Utilization for Cold Region, Heilongjiang University, Harbin 150080, China; 6 Key Laboratory of Molecular Biology of Heilongjiang Province, College of Life Sciences, Heilongjiang University, Harbin 150080, China

**Keywords:** *Lilium pumilum*, PGPB (plant growth-promoting bacteria), *Bacillus cereus*, saline and alkaline stress

## Abstract

Soil salinization severely impacts plant cultivation. *Lilium pumilum* (*L. pumilum*) exhibits tolerance to saline–alkali stresses. One *Bacillus cereus* strain, LpBc-47, possesses the ability of growth promotion and saline–alkali tolerance. The microbial diversity of *L. pumilum* was assessed through metagenomic sequencing. LpBC-47 obtained from *L. pumilum* was subjected to physiological and biochemical analyses and whole-genome sequencing. The effects of endophytic bacteria on plants were evaluated by measuring growth parameters, physiological indices, antioxidant enzyme activities, and ROS content. Microbial diversity analysis revealed that the abundance of endophytic bacteria in *L. pumilum* decreased under saline–alkali conditions, whereas the abundance of *Bacillus cereus* increased. Physiological and biochemical analysis showed that LpBC-47 has the characteristics of promoting growth and reducing plant damage caused by salt–alkali stress, such as phosphorus solubilization, nitrogen fixation, siderophore production, IAA, and ACC deaminase synthesis. Genomic analysis revealed that LpBC-47 contains growth-associated and stress-alleviation genes. GFP indicated the colonization of LpBc-47 in the roots and bulbs of *L. pumilum*. The LpBc-47 inoculant plant increased leaf length and dry weight, elevated proline and chlorophyll levels, enhanced antioxidant enzyme activity, and reduced oxidative damage. This study highlights the potential of LpBc-47 for improving plant growth under saline–alkali conditions.

## 1. Introduction

Land salinization represents a significant challenge to the development of plants. Over 60% of land and 30% of cropland globally have been severely impacted. Saline stress in the soils of Songnen Plain is primarily caused by the presence of NaHCO_3_ [1]. *L. pumilum,* a perennial wild medicinal herb that has the ability to thrive in soils of extremely high salinity, has been traditionally used for its tonic and strong properties [2,3]. *L. pumilum* is an excellent plant material for salinity stress studies due to its strong resistance to disease, cold, and salinity [4]. The improvement of resistance is not solely a consequence of the action of genes. It is likely that other factors are involved, forming a complex system [5]. Microorganisms play a pivotal role in the process of plant growth, and the adaptation of plants to local environments is inextricably linked to microorganisms [6]. Plant endophytic bacteria are a group of bacteria that colonize the interior of plants. They have the ability to accelerate nutrient uptake and utilization, control plant diseases, and cope with abiotic stresses [7].

Endophytic bacteria in lilies were first discovered in 1986 and were found in the bulbs, underground stems, leaves, and young embryos of healthy lilies [8]. Research on *Lijiang lilies* has shown that there are significant differences in the number of bacteria present in the different organs and cells of lilies, and the bacterial content is highest in the cells of the bulb tissue [9]. The diversity of microbial communities in *brown lily* seedlings suggests that endophytic bacteria were the most abundant in lily bulbs [10]. *Burkholderia gladioli*, isolated from the endophytic bacteria of *Longya lily*, has the function of inhibiting the activity of *Botrytis cinerea* [11]. *Burkholderia* sp. *FJb-2*, which was isolated from the *red core lily (Fangio)*, exhibited significant in vitro antibacterial and growth-promoting effects [12]. *Pseudomonas aeruginosa Ld-08,* isolated from *Sichuan lily,* has anti-fungal and growth promoting properties [13]. *Bacillus velezensis Lle-9*, isolated from lilies in Yichang, has antifungal and plant growth-promoting effects [14]; *Bacillus subtilis* has an effect on the growth, yield, ornamental qualities and pigmentation of the lily variety ‘*Savana*’ [15]. The role of endophytic bacteria in promoting growth and improving plant salt tolerance in *L. pumilum* has not been thoroughly analyzed yet.

Endophytic PGPB (plant growth-promoting bacteria) can assist host plants in coping with adversity by modifying root structure and inducing resistance [16]. The bacterium *Bacillus cereus* was first identified in Norway in 1950 as a cause of vomiting [17]. Subsequent investigations have demonstrated that *Bacillus cereus*, when functioning as an endophyte, is capable of assisting host plants in dealing with stress [18]. *Bacillus* has the capacity to fix nitrogen in the air and convert inorganic phosphorus to organic phosphorus, exerting a growth-promoting effect on plants [19]. *Bacillus cereus* is a soil-dominant bacterium [20] that is extremely adaptable to natural conditions and is able to withstand a variety of adverse environments due to its simple nutrient requirements for growth, large size of the individual, and fast growth and reproduction rates [21]. In comparison to normal cells, *Bacillus* exhibits a resistance to heat that is 105 or more times greater, a resistance to ultraviolet and ionic radiation that is more than 100 times greater, and a resistance to chemical disinfectants that is highly elevated [22]. The robust survivability demonstrated by *Bacillus* enables it to thrive in challenging environments. As a plant endophyte, *Bacillus cereus* facilitates plant adaptation to adversity and enhances plant survival under such conditions. A one-unit increase in pH results in a one-thousand-unit decrease in iron activity in solution [23]. Therefore, iron deficiency stress represents another challenge encountered by plants when subjected to salt stress. In contrast, *Bacillus cereus* is capable of secreting iron carriers, which facilitate the uptake of iron ions from the soil, thereby aiding the host plant in combating iron deficiency stress [24]. Further investigation is required to elucidate the underlying mechanism through which *Bacillus cereus* facilitates plant growth and mitigates the impacts of abiotic stress on plant growth.

## 2. Materials and Methods

### 2.1. Plant Material

*L. pumilum* samples were collected from saline–alkali soil (pH = 9.52, Ece = 2430 µS/cm) and normal soil (pH = 7.02, Ece = 143 µS/cm) on the Songnen Plain of Heilongjiang Province in Northeast China. Laboratory-grown *L. pumilum* was cultivated in a controlled-growth chamber at 25 ± 2 °C with a 16 h light/8 h dark cycle.

### 2.2. Microbial Diversity Analysis

Bulbs of *L. pumilum* from saline–alkali soil (pH = 9.52) and normal soil (pH = 7.02) were sent to MajorBio Technology (Shanghai, China) for microbial diversity sequencing, and the results were analyzed.

### 2.3. Screening and Identification of Endophytic Bacteria

Endophytic bacterial strains were isolated from *L. pumilum* bulbs using the dilution–coated plate isolation method [25]. The bulb surfaces were sterilized and cut into small pieces, and the last wash water was retained as a control. If there was no colony growth on the control plate, it was considered clean, the sample was ground in a sterile centrifuge tube, and the suspension was resuspended in sterile water to obtain a bacterial suspension. A total of 200 µL supernatant was spread onto NA solid medium, then inverted in an incubator at 30 °C for 3 days. After the colonies had grown, the 16S rRNA gene was amplified by PCR amplification with the primer 27F (5′-AGAGTTTGATCCTGGCTAG-3′) and 1492R (5′-CTACGGCTACCTTGTTACGA-3′), and the amplified PCR product bands (~1540 bp for 16S rRNA sequences) were commercially sequenced (Kumei Biotechnology Co., Ltd., Changchun, China). The sequenced results were compared to those in the GenBank database using the BLAST (https://blast.ncbi.nlm.nih.gov/Blast.cgi?PROGRAM=blastn&PAGE_TYPE=BlastSearch&LINK_LOC=blasthome, accessed on 15 April 2025) algorithm (National Center for Biotechnology Information, Bethesda, MD, USA).

### 2.4. Screening of Bacteria with Growth-Promoting and Saline–Alkali Tolerance

To assess bacterial resistance to salinity, solid LB medium with varying concentrations of NaHCO_3_ (0 mM, 100 mM, 200 mM, 300 mM, 400 mM, 500 mM, 600 mM) was prepared. The obtained endophytes of *L. pumilum* were activated in 1 mL liquid LB medium until the OD_600_ reached 0.8, and 2.5 µL was inoculated onto the medium and incubated at 30 °C.

*L. pumilum* seeds were washed and placed on 1/2 MS medium or 1/2 MS medium with 30 mM NaHCO_3_. A total of 100 µL endophytic bacteria (OD_600_ = 0.8) was streaked on the lower part of the medium. Plates were incubated at 25 °C with 16h light daily for 10 days. Three sets of replicated experiments were conducted.

### 2.5. Physiological and Biochemical Identification of LpBc-47

Inoculate 10 µL endophytic bacteria (OD_600_ = 0.8) into 5 mL tyrosine decarboxylase test tube and the amino acid decarboxylase control tube (without amino acids) for tyrosine decomposition experiment, each covered with 20 µL of sterile paraffin oil, incubate at 37 °C for 1–4 days, and observe the results every day. If the experimental group exhibits a purple coloration, it is considered positive [26].

Inoculate 5 µL experimental strains (OD_600_ = 0.8) into the starch agar medium for the hydrolyzed starch experiment, take out the plate after culturing at 30 °C for 1–3 days, and drop 50 µL of Lugol’s iodine solution on the plate. Observe whether there is a colorless transparent circle in the culture medium around the colony [27].

Take 2 mL activated bacteria solution (OD_600_ = 0.8), add 2 mL of 40% NaOH, add 0.5 mg creatine, vortex for 30 min, turn red, and record as positive for VP experiments. 

MR experiments were performed using the methyl red chromogenic method, which turned red and was recorded as positive [28].

### 2.6. The Whole Genome Sequencing of LpBc-47

We sent the selected strain 13 to MagiGene Biotechnology Company (Guangzhou, China) for whole genome sequencing. The PE150 sequencing of the constructed amplicon libraries was performed using Illumina or MGI platforms (Guangdong Magigene Biotechnology Co., Ltd., Guangzhou, China). The prediction of genome components includes the prediction of coding genes, repeat sequences, noncoding RNA, and prophages. Genome-wide Blast searches were performed on the following databases: NR (Non-Redundant Protein Database), Swiss-Prot, GO (Gene Ontology), KEGG (Kyoto Encyclopedia of Genes and Genomes), COG (Clusters of Orthologous Groups), etc. to predict gene function.

### 2.7. Growth Conditions and Growth Promoting Characteristics of LpBc-47

To evaluate the saline–alkali tolerance of LpBc-47, 10 µL LpBc-47 bacterial solution (OD_600_ = 0.1, 8 × 10^7^ CFU/mL) was added to 10 mL sterile LB liquid medium. Subsequently, the medium was supplemented with 0 mM, 100 mM, 200 mM, 300 mM, 400 mM, 500 mM, or 600 mM NaHCO_3_ to determine the growth curves under alkali stress. The inoculated tubes were placed on a shaking table and incubated with shaking at 30 °C and 130 rpm; 200 µL samples of the bacterial solution were taken at hourly intervals, and the absorbance at OD_600_ was measured within 18 h.

2.5 µL LpBc-47 (OD_600_ = 0.8) was inoculated on the solid LB medium with a working concentration of 0–600 mM NaHCO_3_, the culture was then incubated at 30 °C upside down to observe the growth of LpBc-47 under saline alkali conditions.

To estimate the maximal growth and minimal inhibitory pH value, add 10 µL LpBc-47 (OD_600_ = 0.1, 8 × 10^7^ CFU/mL) liquid to 10 mL sterilized LB liquid medium added with different pH values (3–10) and mix well to cultivate the bacterial isolates in a shaker at 30 °C with constant shaking at 130 rpm. The absorbance value at OD_600_ was measured using an enzyme-linked immunosorbent assay (ELISA) reader every hour and measured continuously for 24 h. The experiments were performed in triplicate.

The bacterial growth-promoting characteristics were explored with the configured cellulose decomposing microorganism medium for detecting cellulose decomposition [29]. The presence or absence of phosphorus-solubilizing ability of bacteria was detected using the NBRIP medium [30]. Ashby nitrogen-free medium was used for testing nitrogen fixation capacity [31]. The ability of bacteria to solubilize potassium was tested using a silicate medium [32]. Iron carrier generating capacity was detected with CAS solid medium [33]. The production capacity of IAA was detected by the Salkowski colorimetry method [34], and the ACC deaminase was determined using DF (thiamine nitrogen source) and ADF (ACC nitrogen source) media [35]. A total of 2.5 µL LpBc-47 (OD_600_ = 0.8) was inoculated onto the medium.

### 2.8. Construction of GFP-Labelled LpBc-47

The method for preparing product LpBc-47-competent cells was modified according to the method of Guo et al. [36]. Newly activated colonies were inoculated into 1 mL of LB liquid medium, incubated overnight at 37 °C, and shaken at 180 rpm. The cultures were injected into freshly prepared 100 mL LB liquid medium and shaken at 180 rpm for 3–5 h until the OD_600_ reached 0.8. Subsequently, the bacterial solution was subjected to an ice bath for 20 min and centrifuged at 7000 rpm for 10 min at 4 °C for the collection of bacteria. Thereafter, the cells were washed three times with sterile water, centrifuged at 7000 rpm for 10 min at 4 °C, and then washed once with 10 mL of precooled 75 mM CaCl_2_. The precipitates were gathered and resuspended in 0.7 mL of CaCl_2_ and 0.3 mL of 50% glycerol. Electrically competent LpBc-47 bacteria were thereby obtained. One µg of the pHAP-II plasmid was added and gently mixed with the competent cells. This mixture was then transferred to a precooled 1 mm electroexcitation cup and subjected to an ice bath for 10 min. Electroexcitation was carried out at 1200 V, and the bacterial suspension was retrieved into a centrifuge tube by adding 1 mL of LB liquid medium and incubated at 130 rpm for 3 h at 37 °C. Finally, the bacterial suspension was plated on LB plates with kanamycin resistance and incubated at 37 °C overnight to screen for the transformants containing pHAP-II. Positive colonies were selected for further verification of the genotype by 16 sRNA gene sequencing. Seedlings co-cultured with LpBc-47 for 7 days under normal conditions and under NaHCO_3_ stress were chosen for observation of GFP fluorescence.

### 2.9. LpBc-47 Promotes the Growth of L. pumilum and Improves Its Salt Tolerance

LpBc-47 was cultured overnight in LB liquid medium to OD_600_ at 0.8. The bacteria were collected by centrifugation, and the pellet was resuspended in an equal amount of sterile water to obtain a bacterial suspension. The experiment was divided into four groups. The first group of plants was irrigated in the pots only with 50 mL water (non-saline, as the control); the second group of plants was irrigated with 50 mL resuspended LpBc-47 solution; the third group of plants was irrigated with 50 mL water for 5 days (maintain consistency with the fourth group salt treatment time), then the pot was irrigated with 50 mL saline solution of 50 mL NaHCO_3_; and the fourth group of plants was inoculated with 50 mL LpBc-47 solution for 5 days, then irrigated with 50 mL saline solution of 50 mL NaHCO_3_.

To investigate the effect of LpBc-47 on *L. pumilum* under salt stress, the leaves of non-stressed *L. pumilum* were employed as controls, and the E+, E+ASS, and E−ASS groups were utilized for DAB and NBT staining. DAB staining was adopted to detect the active site of peroxidase in cells. The treated leaves were excised and stained in the dark by immersion in 1% DAB solution at 30 °C for 24 h, followed by the removal of chlorophyll with 95% ethanol for result observation [37]. NBT staining was employed in living plant tissues to detect superoxide anions, which are oxygen-containing free radicals capable of reducing NBT and generating insoluble blue formamide compounds. After staining, areas where superoxide anions accumulate presented as blue to dark blue. The treated leaves were immersed in 0.02% NBT solution for 12 h at room temperature, and chlorophyll was eliminated with 95% ethanol [38].

The proline content in plants was determined by the acid ninhydrin method [39], the spectrophotometric detection of hydrogen peroxide (H_2_O_2_) and superoxide anions (O_2_^−^) [40]. The MDA was then identified by the thiobarbituric acid method [41], POD activity was determined by the guaiacol oxidation method [42], and the detection of SOD was conducted through the ascorbic acid method [43]. The CAT was identified through the use of spectrophotometry [44], and chlorophyll content was determined using a chlorophyll meter, SPAD-502 Plus (Konica Minolta, Tokyo, Japan).

## 3. Results

### 3.1. Microbial Diversity Analysis

**Figure 1:**
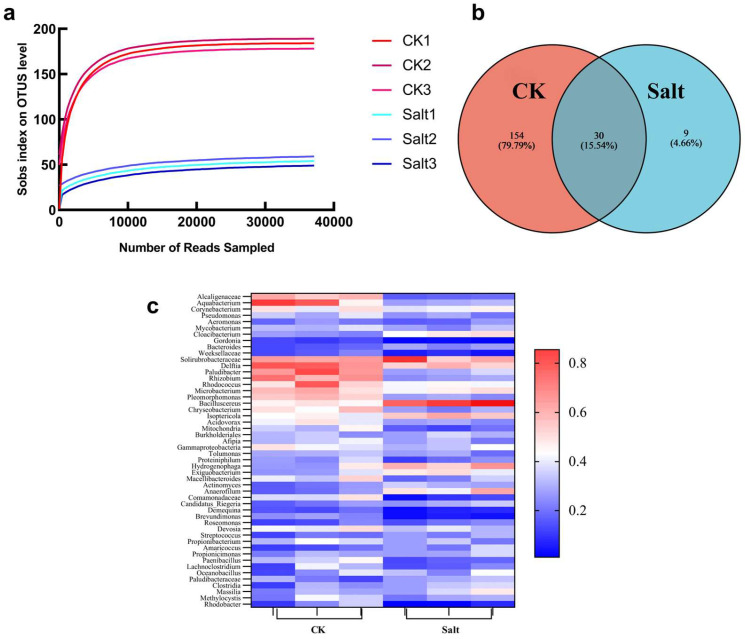
Microbial diversity and analysis of common microorganisms before and after salt stress. (**a**) Microbial diversity curves of non-salt-stressed and salt-stressed *L. pumilum* seedlings. (**b**) Venn diagram showing the number of microorganisms shared by non-salt-stressed and salt-stressed groups, which was 30 species. (**c**) Analysis of endophytic bacterial abundance in *L. pumilum* under saline–alkali and unstressed soil conditions. The red indicates an increase in bacterial abundance, whereas blue indicates a decrease.

The curve of the sequencing results revealed a notable decline in the microbial diversity of *L. pumilum* under saline stress (Figure 1a). The findings indicated a significant difference in the quantity of endophytic bacteria present in *L. pumilum* cultivated under saline soil conditions compared to non-stress soil conditions. Saline stress had significantly altered the microbial community structure of the *L. pumilum* bulb. The Venn diagram (Figure 1b) revealed that a total of 184 endophytic bacteria were isolated from *L. pumilum* growing in non-stressed soil conditions. In contrast, only 39 endophytic bacteria were isolated under saline–alkali conditions, among which 30 were also identified in the non-stressed soil. Nine new bacterial species were exclusively isolated under saline–alkali stress conditions. The heat map shows the quantitative analysis of the 30 coexisting endophytic bacteria in saline–alkaline soil and non-stressed soil (Figure 1c). Red indicates a higher abundance of endophytic bacteria in a given soil condition, while blue indicates a lower abundance. The data reveal that 23 endophytic bacteria had significantly lower abundance in saline–alkali soil, suggesting that salt stress negatively impacts their survival. Conversely, seven endophytic bacteria were more abundant in saline–alkaline soil, implying their potential to adapt to such conditions and assist plants in stress resistance. Notably, *Bacillus cereus* showed a marked increase in saline–alkali conditions.

### 3.2. Screening and Identification of Endophytic Bacteria

A total of 24 different types of endophytic bacteria were identified by 16S rRNA sequencing following the screening of *L. pumilum* endophytes on NA medium. The results are presented in Table 1.

### 3.3. Screening of Bacteria with Growth-Promoting and Saline–Alkali Tolerance

**Figure 2:**
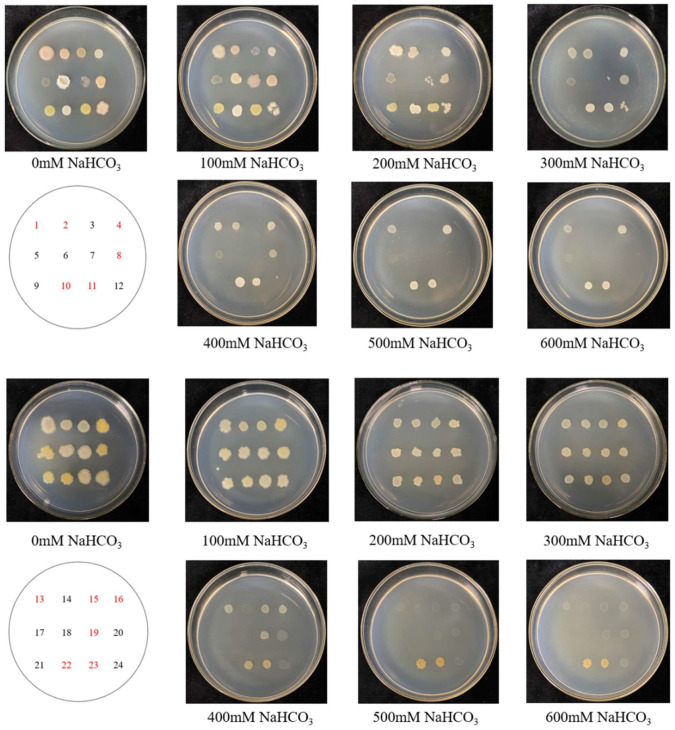
Growth of endophytic bacteria under NaHCO_3_-stressed strains 1–12 and strains 13–24 on saline–alkali stress gradient medium. Twelve strains grew well on LB solid medium with 400 mM NaHCO_3_.

Twenty-four endophytic bacteria isolated from *L. pumilum* were tested for salt–alkali tolerance. Twelve of the twenty-four endophytic bacteria exhibited normal growth on LB solid medium with 400 mM NaHCO_3_ (pH = 8.71) (Figure 2), showing they have salt–alkali tolerance.

**Figure 3:**
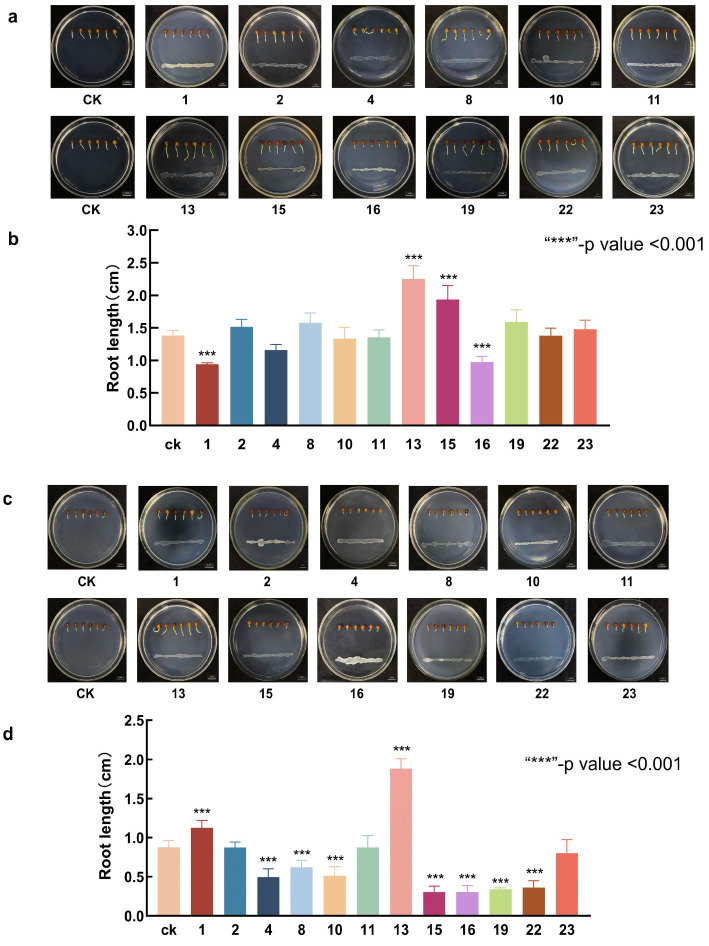
Growth of *L. pumilum* in co-culture with endophytic bacteria. (**a**) Seed germination status of *L. pumilum* in 1/2MS medium. (**b**) Root length of *L. pumilum* seeds after 10 days of growth. (**c**) Seed germination status of *L. pumilum* in 1/2MS medium with 30 mM NaHCO_3_. (**d**) Root length of *L. pumilum* seeds after 10 days of germination under 30 mM NaHCO_3_ saline–alkali stress.

Endophytic bacteria exhibiting a certain degree of salt tolerance were co-cultured with *L. pumilum* seeds. The experiments were conducted on two distinct media: the first was the standard 1/2 MS medium; the second was the 1/2 MS medium with 30 mM NaHCO_3_ stress. Bacteria that exhibited both salt tolerance and the capacity to promote *L. pumilum* seed growth under both conditions were isolated. After the seeds were inoculated with endophytic bacteria and cultivated for 10 days, distinct growth trends were witnessed (Figure 3a). The majority of the endophytic bacteria were capable of promoting the growth of *L. pumilum*. In all the replicated experiments, all seeds were able to germinate, suggesting that endophytic bacteria did not have an influence on the germination rate of seeds under these circumstances. The experimental results can be presented as a bar chart to observe more intuitively the effect of endophytic bacteria inoculation on the growth of *L. pumilum* (Figure 3b). In contrast to the control group, strains No. 1, No. 4, and No. 16 inhibited the germination of *L. pumilum*, and strain 13 had the most favorable growth promotion effect.

When incubated in 1/2MS medium at a working concentration of 30 mM NaHCO_3_ for 10 days (Figure 3c). In the bar chart (Figure 3d), it can be noted that the roots of *L. pumilum* in the control group grew more slowly than those in the non-stress condition, indicating that the growth rate of *L. pumilum* would be affected by the saline–alkali environment. In the experimental group, most endophytes showed an inhibitory tendency towards *L. pumilum*. The growth of bacteria No. 2 and 11 was essentially similar to that of the control group, and only endophytic bacteria No. 1, 13, and 23 could promote the growth of *L. pumilum*. The most efficacious effect of growth promotion was evident in strain 13. Strain 23 exhibited the capacity to withstand 600 mM NaHCO_3_ (Figure 2) and to promote root elongation in *L. pumilum* under stress conditions. However, in the absence of stress, the root elongation of *L. pumilum* treated with strain 23 was comparable to that of the control group. In evaluating the growth-promoting effects under both non-stress and stress conditions, strain 13 demonstrated a significant enhancement in root elongation of *L. pumilum* in both scenarios. Strain 13 had a stronger growth promotion effect than strain 23, and it also had a strong salinity tolerance (400 mM NaHCO_3_). Consequently, strain 13 was selected for further investigation.

### 3.4. Identification of Bacteria

**Figure 4:**
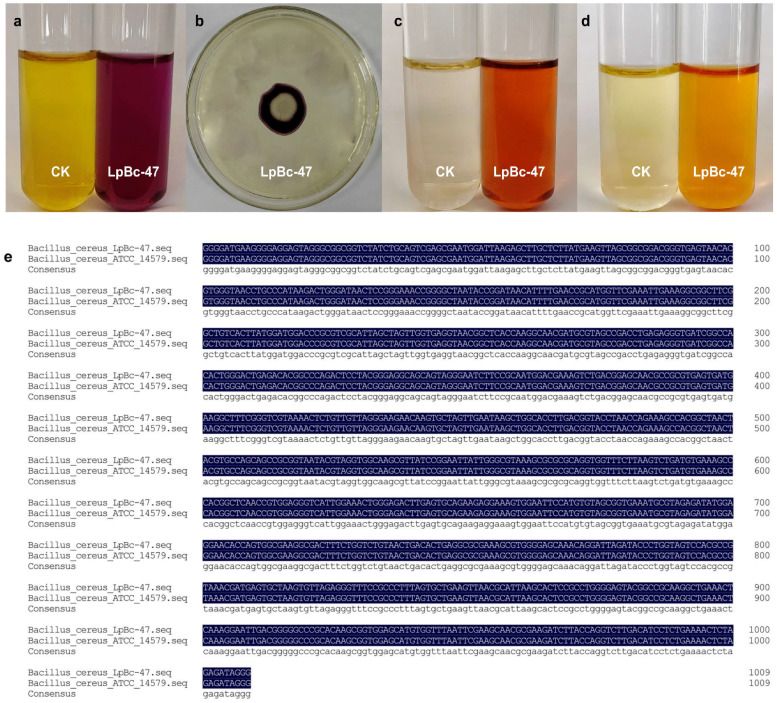
Physiological and biochemical tests of LpBc-47. (**a**) Amino acid decarboxylase assay: the control group was yellow and the experimental group purple, indicating LpBc-47 can decompose tyrosine. (**b**) Starch hydrolysis enzyme assay: transparent circles around colonies indicate LpBc-47 can break down starch. (**c**) Voges–Proskauer (VP) test for acid production: the red experimental group indicates that LpBc-47 can break down glucose to produce acid. (**d**) Methyl red (MR) test for producing acid reaction: the red experimental group indicates that LpBc-47 can produce acid and reduce pH. (**e**) Blast with standard bacterial strain in *BERGEY’S MANUAL*.

Strain 13 can promote the seed germination and enhance the salt and alkali tolerance of *L. pumilum*. The bacteria possess the capacity to decompose tyrosine (Figure 4a) and hydrolyze starch (Figure 4b). The V-P and MR experiments yielded positive outcomes (Figure 4c,d). The results of the 16S rRNA sequencing and the physiological and biochemical characteristics mentioned in the Berger manual book indicated that strain 13 is identified as *Bacillus cereus* (GenBank accession number: SAMN05506965).

In conjunction with the assessment of microbial diversity, the numbers of *Bacillus cereus* in the *L. pumilum* cultivated in a saline–alkali environment was markedly elevated in comparison to the *L. pumilum* grown in the no-saline environment (Figure 1c). This observation suggests that under saline–alkali conditions, *Bacillus cereus* may confer resilience to plants. Strain 13 was sent to MagiGene Biotechnology Company (Guangzhou, China) for genome sequencing. Average nucleic acid similarity (ANI) is one of the most powerful indicators of the distance between bacterial genomes. The evolutionary distance of genome quality inspection is reflected based on the average of the comparison of all orthologous protein sequences of genome quality inspection. When ANI > 95%, it means that two genomes belong to the same species. The sequencing results showed that the ANI value of strain 13 and *Bacillus cereus strain FORC_047* reached 96.1856, The analysis revealed that the bacterial isolate corresponds to *Bacillus cereus ATCC_14579* (standard bacterial strain in *BERGEY’S MANUAL*) with 100% identity (Figure 4e). Therefore, strain 13 was identified as *Bacillus cereus*. The bacterium was obtained from *L. pumilum* and was identified as belonging to the *Bacillus cereus strain FORC_047*. It was therefore named LpBc-47.

### 3.5. Genomic Analysis of LpBc-47

The sequencing results for LpBc-47 revealed the acquisition of a total of 9,153,766 read fragments, with a collective length of 5.7 Mbp and a GC content of 34.91%; a total of 5750 protein-encoding genes and 120 non-coding genes were predicted. The mean length of protein-coding genes is 835 base pairs (bp), with a considerable number of them exerting a beneficial influence on plant growth and stress resistance. These includes 94 genes involved in nitrogen compound metabolism and 30 genes associated with phosphorus metabolism, among others. Additionally, eighty-eight genes are associated with potassium ion transport, six genes are linked to indoleacetic acid synthesis, ten genes have been demonstrated to enhance antioxidant activity in plants, and one hundred and ninety genes are involved in iron ion binding in this category (Table 2). Only a few representative gene names are listed in the Table 2.

**Figure 5:**
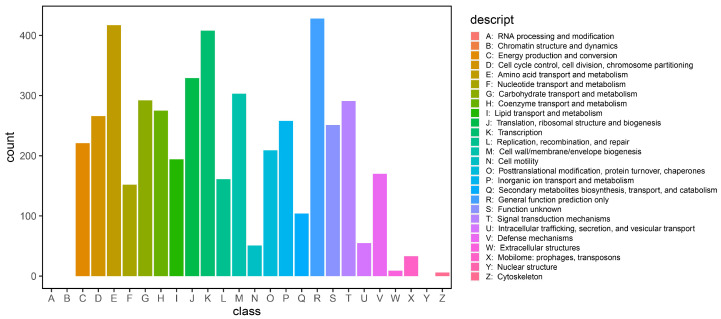
Results of COG analysis of LpBc-47 genome COG analysis showed that the LpBc-47 genome had the most genes related to amino acid transport and metabolism, transcription, and growth-promoting characteristics of LpBc-47.

COG annotation is the process of inferring the functions of unknown proteins based on the annotations of known proteins. The analysis of LpBc-47 (Figure 5) revealed that the majority of the proteins were related to E (amino acid transport and metabolism), K (transcription) and R (general function prediction only). Additionally, a considerable number of proteins involved in T (signal transduction mechanisms), J (translation, ribosomal structure and biogenesis), M (cell wall/membrane/envelope biogenesis) and G (carbohydrate transport and metabolism) are instrumental in LpBc-47 survival, enhancing their viability under saline–alkali conditions and aiding host plants in resisting stress.

**Figure 6:**
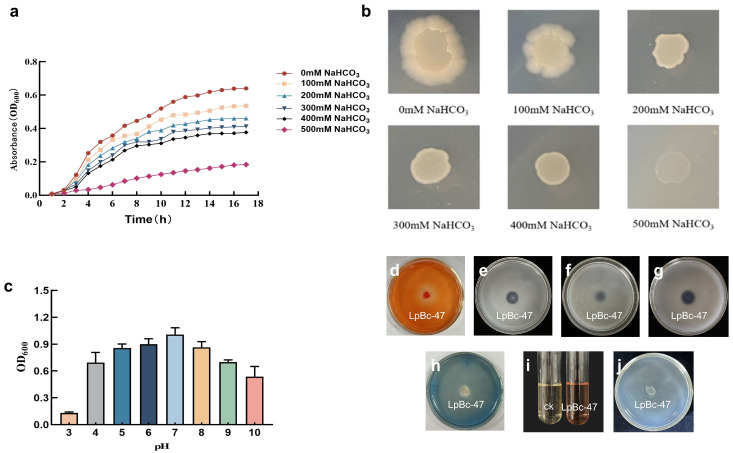
Bacterial characterization. (**a**) Growth curves of LpBc-47 under different concentrations of NaHCO_3_ stress in the LB liquid medium. (**b**) Growth states of LpBc-47 under different concentrations of NaHCO_3_ stress in the LB solid medium. (**c**) Growth curves of LpBc-47 at different pH. (**d**) The ability to dissolve cellulose was assessed on CDMM medium. (**e**) The capacity of phosphorus solubilization was measured on NBRIP medium. (**f**) The ability of fix nitrogen was assessed on Ashby nitrogen-free medium. (**g**) The capacity to decompose potassium was measured on silicate medium. (**h**) The capacity of produce siderophore was measured on CAS medium. (**i**) The capacity to produce IAA was measured used Salkowski’s reaction. (**j**) The capacity to produce ACC deaminase was measured on ADF medium.

In the LB liquid medium, the growth curve of the bacteria exhibited a consistent increase, with accelerated growth between 3 and 11 h and a subsequent stabilization after 11 h. Furthermore, an increase in stress concentration resulted in a decline in the growth rate. As the concentration of NaHCO_3_ increased, the growth curve of the bacterial solution became lower. The growth rate was found to be markedly slow in the presence of 500 mM NaHCO_3_ (Figure 6a). In the LB solid medium, the growth of bacterial solution was increasingly inhibited as the concentration of NaHCO_3_ increased. LpBc-47 exhibited normal growth on LB medium containing 400 mM NaHCO_3_. However, at 500 mM NaHCO_3_, the growth rate was markedly reduced, and the bacterial growth was significantly inhibited (Figure 6b).

The LpBc-47 were incubated at varying pH (3–10) conditions for 24 h, and the absorbance was measured at OD_600_. The results demonstrated that the highest OD_600_ value and optimal activity were observed at pH = 7. As alkalinity increased, the growth rate of LpBc-47 declined. Bacterial growth was severely inhibited in an acidic environment with a pH value of 3 (Figure 6c). In CDMM medium, hyaline rings were produced around the colonies, thereby demonstrating that the LpBc-47 was capable of decomposing cellulose (Figure 6d). In NBRIP medium, transparent rings were produced around the colonies, thereby demonstrating that the LpBc-47 is capable of dissolved phosphorus (Figure 6e). In Ashby nitrogen-free medium, a transparent circle appeared around the LpBc-47, thereby demonstrating its capacity to fix nitrogen from the atmosphere for its own growth and development (Figure 6f). A transparent circle surrounded the LpBc-47 colony on Silicate bacterial culture medium, indicating its ability to decompose inorganic phosphorus, making insoluble inorganic phosphorus in the soil available for plant absorption and utilization (Figure 6g). The color of the medium surrounding the endophyte LpBc-47 altered from blue to red, and iron carrier secretion circles emerged, indicating that LpBc-47 is capable of producing iron carriers (Figure 6h). The Salkowski chromogenic method was employed to ascertain whether the endophyte produced IAA; the results demonstrated that the LB supernatant of the group containing the LpBc-47 exhibited a markedly redder hue compared with the control group, thereby substantiating the production of IAA (Figure 6i). LpBc-47 is capable of growing on ADF medium with ACC serving as the sole nitrogen source. This finding suggests that LpBc-47 has the ability to decompose ACC, which is the precursor of ethylene. By doing so, it can prevent excessive ethylene accumulation under stress conditions, thereby assisting plants in alleviating salt stress (Figure 6j).

### 3.6. LpBc-47 Promotes the Growth of L. pumilum and Improves Plant Salt Tolerance

**Figure 7:**
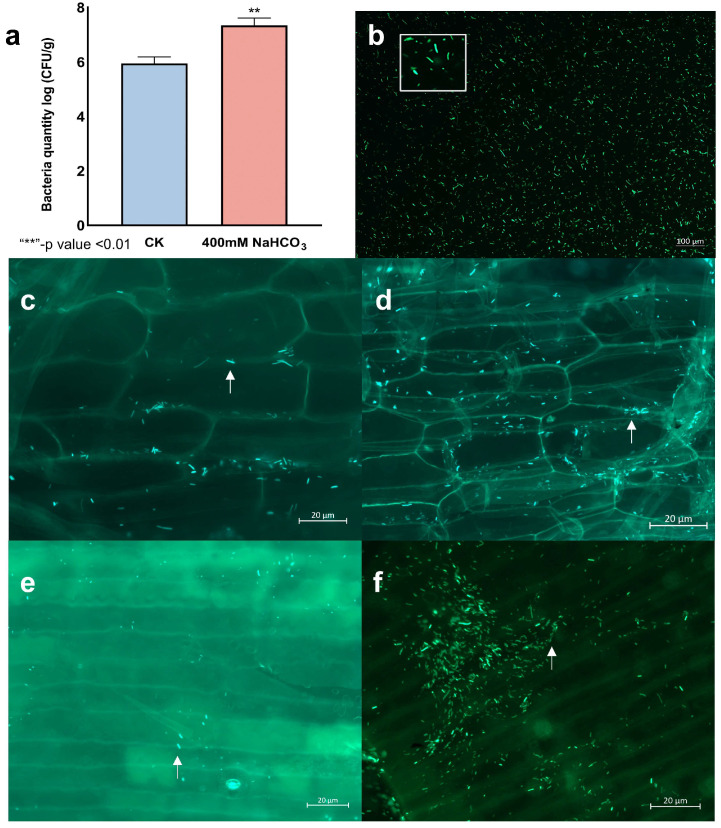
Colonization of LpBc-47 in *L. pumilum* roots and bulbs. (**a**) Quantification of LpBc-47 on *L. pumilum* root and bulbs under normal and stress conditions using dilution separation methods. (**b**) The morphology of LpBc-47. (**c**) The colonization of root with GFP-expressing LpBc-47 under non-stress conditions. (**d**) The colonization of root with GFP-expressing LpBc-47 under 400 mM NaHCO_3._ (**e**) The colonization of bulb with GFP-expressing LpBc-47 under non-stress conditions. (**f**) The colonization of bulb with GFP-expressing LpBc-47 under 400 mM NaHCO_3._ The white arrow indicates LpBc-47 colonized in plant tissues, and the white box represents the enlarged LpBc-47.

We quantified the capacity of LpBc-47 to colonize the roots and bulbs of *L. pumilum* by enumerating colony-forming units (CFU). It was found that under 400 mM NaHCO_3_ stress, the colonization of LpBc-47 in *L. pumilum* was higher than that without stress (Figure 7a). Competent cells of LpBc-47 were constructed and transferred into the green fluorescent protein plasmid pHAP-II (Figure 7b). LpBc-47 colonized the roots of *L. pumilum* under no stress (Figure 7c) or under stress (Figure 7d) and the bulbs under no stress (Figure 7e) or under stress (Figure 7f). Following stress exposure, the number of LpBc-47 colonizing the roots (Figure 7d) and bulbs (Figure 7f) increased significantly.

**Figure 8:**
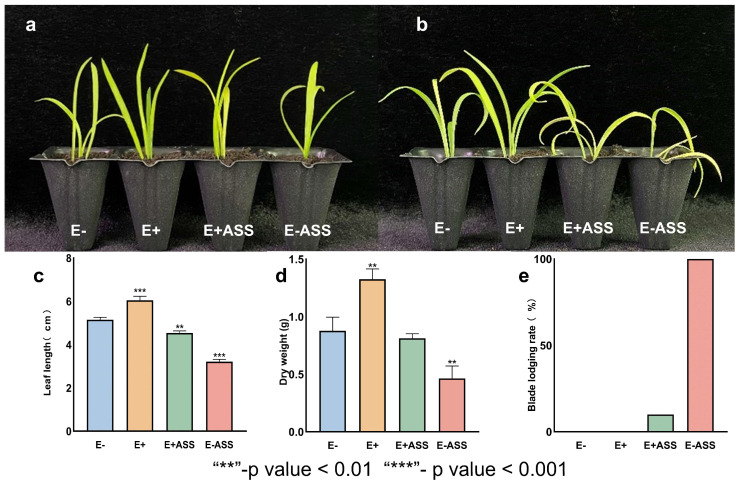
Effect of LpBc-47 on *L. pumilum* plants. (**a**) Before the treatment of saline–alkali stress, four *L. pumilum* seedlings exhibiting consistent leaf number and height were selected for the experiment. LpBc-47 was applied to the E+ and E+ASS groups. (**b**) The E+ASS and E−ASS groups of *L. pumilum* seedlings were subjected to 400 mM NaHCO_3_. The growth of *L. pumilum* seedlings treated with LpBc-47 was observed to be superior to that of the control group, which was not treated with LpBc-47. (**c**) The leaf length of the E+ group exceeded that of the E− group, and the E+ASS group’s leaf length was greater than that of the E−ASS group. (**d**) The dry weight of group E+ was heavier than that of group E−, and the dry weight of group E+ASS outperformed that of group E−ASS. (**e**) Lodging rate of *L. pumilum* seedlings after treatment; all blades in the E−ASS group lodged, while only 10% of blades in the E+ASS group experienced lodging.

In order to explore the effect of LpBc-47 on the growth of *L. pumilum* seedlings and the effect of LpBc-47 on the growth of *L. pumilum* seedlings under salt stress, the seedling irrigation experiments were carried out. The seedlings with the same growth conditions were divided into four groups: E−: no treatment for *L. pumilum* seedlings; E+: LpBc-47 treatment for *L. pumilum* seedlings; E+ASS: LpBc-47 treatment and application of 400 mM NaHCO_3_; E−ASS: application of 400 mM NaHCO_3_ only (Figure 8a). The experiment was repeated three times, and phenotypic observation was carried out 17 days after the treatment (Figure 8b). The physiological indicators related to the growth of *L. pumilum* seedlings were measured. The leaf length of the E+ group was 17% longer than that of the E− group, and the leaf length of the E+ASS group was 41% longer than that of the E−ASS group (Figure 8c), suggesting that the LpBc-47 treatment facilitated the growth of *L. pumilum*. The dry weight of the E+ group was 45% greater than that of the E− group. The weight of the E+ASS group was 75% higher than that of the E−ASS group (Figure 8d), which indicated that LpBc-47 could not only promote *L. pumilum* growth but also reduce the damage of saline stress to *L. pumilum* and minimize the loss of plant biomass. The leaf lodging rate of *L. pumilum* was calculated. After salt stress, *L. pumilum* was damaged and all its leaves were lodging. However, only 10% of the leaves in the E+ASS group were lodging, which was significantly better than those in the E−ASS group (Figure 8e). This indicates that the application of LpBc-47 greatly assisted *L. pumilum* seedlings in alleviating the damage caused by salt stress. The plant length of the E+ group was observed to be greater than that of the E− group, which indicated a growth-promoting effect of LpBc-47. However, following the application of salt stress, the E−ASS group, which had not undergone LpBc-47 treatment, exhibited severe poisoning and pronounced leaf wilting. In contrast, the E+ASS group, which had been treated with LpBc-47, demonstrated relatively robust performance, with less evident leaf wilting. The leaf length and dry weight of the E−ASS group were lower than those of the E+ASS group treated with LpBc-47 (Figure 8c,d), The E−ASS group without LpBc-47 exhibited wilted and collapsed leaves due to salt stress, while only 10% of the leaves in the E+ASS group wilted (Figure 8e). This suggests that increasing the number of LpBc-47 through irrigation can facilitate plant growth and enhance resilience to salinity and alkali stress.

### 3.7. Measurement of Physiological Indexes After Bacteria Treatment Under Salt Stress

**Figure 9:**
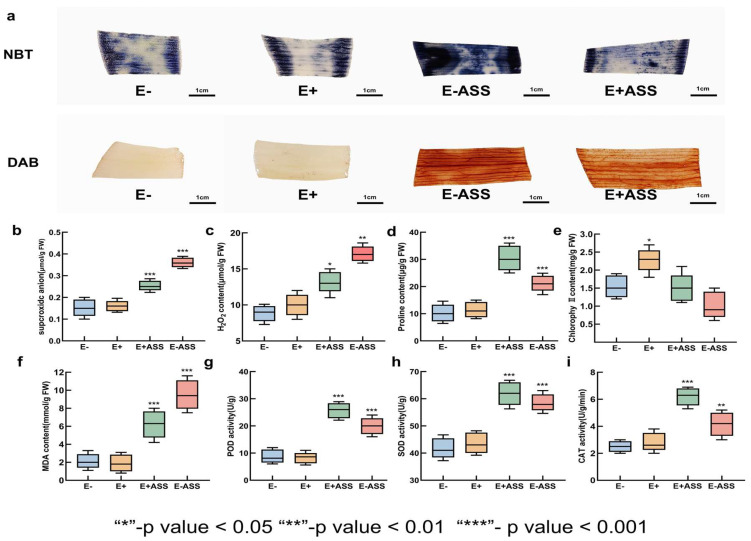
Determination of physiological indexes of *L. pumilum* under salt stress: (**a**) DAB and NBT staining of *L. pumilum* leaves, (**b**) content of O_2_^−^, (**c**) content of H_2_O_2_, (**d**) content of chlorophyll, (**e**) content of MDA, (**f**) content of PRO, (**g**) activities of POD, (**h**) activities of SOD, (**i**) activities of CAT.

By treating the leaves of *L. pumilum* (Figure 9a) with nitroblue tetrazolium (NBT) stainin (blue pigment) and diaminobenzidine (DAB) staining (brown pigment), it was observed that the E−ASS group subjected to 400 mM NaHCO_3_ stress exhibited deeper dark blue and dark brown staining compared to the unstressed E− group. Nevertheless, the color of the E+ASS group with LpBc-47 was paler than that of the stress group without LpBc-47. The E+ groups treated with LpBc-47 exhibited less oxidative damage and presented a lighter staining compared to the E− groups. These findings indicated that NaHCO_3_ treatment significantly elevated the levels of ROS in leaves during seedling growth, while LpBc-47 could decrease the ROS content. The superoxide anion content (Figure 9b) and H_2_O_2_ content (Figure 9c) of the E−ASS group increased markedly under conditions of salt stress, whereas that of the treatment involving LpBc-47 increased to a lesser extent. The results demonstrated that LpBc-47 was capable of alleviating the toxic effects of salt stress on plants. The physiological parameters of *L. pumilum* plants irrigated with bacteria and stress were measured in order to elucidate the mechanism by which LpBc-47 enhances the salt tolerance of its host plant. The application of saline stress resulted in a notable decline in chlorophyll content. The plants subjected to E+ASS exhibited a reduction in chlorophyll levels relative to the E+ group, yet the levels remained comparable to those observed in the E− group (Figure 9d). The concentrations of MDA were found to be significantly lower in the E+ASS group than in the E−ASS group (Figure 9e). In the absence of stress, the concentration of proline in plants is typically low. However, when plants are subjected to saline stress, the proline content increases significantly. The highest proline content was observed in the E−ASS group, while the proline content in the E+ASS group was significantly lower than that in the E−ASS group, indicating that LpBc-47 can assist plants in mitigating saline stress (Figure 9f). POD is the primary antioxidant enzyme in the reactive oxygen scavenging system, which enables plants to avoid or alleviate reactive oxygen damage. After stress, the POD enzyme activity in the plants rose, and that of the E+ASS group treated with LpBc-47 was 31% higher than that of the E−ASS group (Figure 9g). After stress, the SOD activity of the E−ASS group rose rapidly, but with the assistance of LpBc-47, the SOD activity of the E+ASS group was higher (Figure 9h). POD can eliminate H_2_O_2_ in plants. CAT, SOD, and POD act in synergy to assist plants in alleviating the damage caused by stress. After stress, the CAT activity of the E+ASS group is 53% higher than that of the E−ASS group (Figure 9i). It can be observed that LpBc-47 can enhance the activity of plant antioxidant enzymes and enable plants to better resist salt stress.

**Figure 10:**
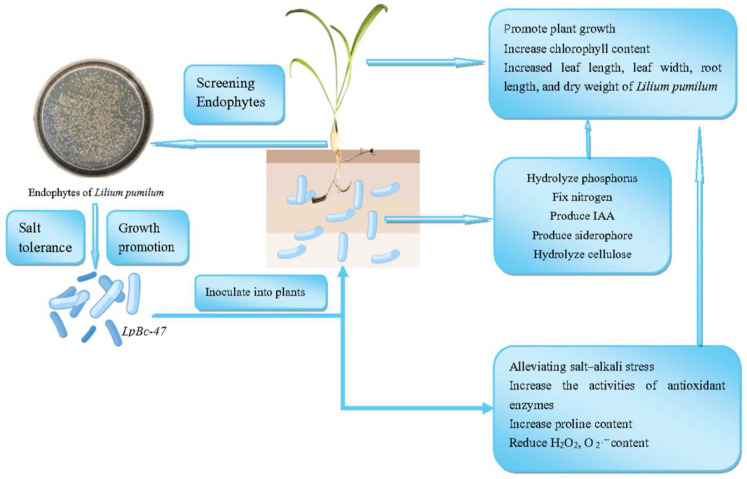
Model diagram of *Bacillus cereus* LpBc-47 promoting the development of *L. pumilum* and enhancing *L. pumilum* saline–alkali tolerance.

Endophytic bacteria isolated from *L. pumilum* have the ability to promote plant growth and alleviate saline–alkali stress. The screened endophytes, especially strain LpBc-47, exhibit multiple advantages: they augment chlorophyll content, enhance morphological traits (leaf length, leaf width, root length, and dry weight), and display plant growth-promoting activities such as phosphorus hydrolysis, nitrogen fixation, IAA production, siderophore synthesis, and cellulose degradation. Under salt–alkali stress, these endophytes alleviate oxidative damage by increasing antioxidant enzyme activity and proline accumulation while reducing detrimental reactive oxygen species (H_2_O_2_ and O_2_^−^). The findings emphasize the potential of these microbial symbionts in sustainable agriculture and stress-resistant crop cultivation (Figure 10).

## 4. Discussion

*L. pumilum* is a plant with a high salt tolerance. Current research regarding its salt tolerance centers on salt tolerance genes and transcription factors. However, the remarkable salt tolerance of this plant is not merely attributed to the expression of salt tolerance genes but also works synergistically with its internal endophytic bacteria to enhance its tolerance to abiotic stress [45], so the endophytic bacteria of *L. pumilum* have important research value. *Paenibacillus polymyxa SK1*, isolated from *Lilium lancifolium*, and *Bacillus stratosphericus LW-03*, isolated from the bulbs of *Lilium wardii*, were demonstrated to have pro-growth characteristics [46,47]. *Bacillus halotolerans LBG-1–13*, isolated from *Lilium (davidii var unicolor*), is capable of promoting plant growth and enhancing salt tolerance [48].

In order to verify the characteristics of *L. pumilum*’s promotion of growth and improvement of plant salt tolerance, in this study, 24 strains of endophytic bacteria were isolated from *L. pumilum* growing in saline–alkali soil, and their salt tolerance was examined. Twelve endophytic bacteria strains capable of tolerating 400 mM NaHCO_3_ were obtained. The bacterial strain LpBc-47 was acquired, which could significantly enhance seed germination and root elongation of *L. pumilum*.

Abiotic stress can exert an influence on the diversity and richness of plant rhizosphere microorganisms and endophytic bacteria, and the diversity of endophytic bacteria declines after stress [49,50], but the number of beneficial microorganisms will increase significantly. This phenomenon is characterized as a “call for assistance” from plants to microorganisms [51]. Figure 1c illustrates that the population of endophytic bacteria was altered under saline–alkali stress conditions. Microbial diversity analysis revealed that among the thirty endophytic bacterial species present both before and after salt stress, seven exhibited an increase in abundance. Notably, the populations of *Bacillus cereus*, *Cloacibacterium*, and *Hydrogenophaga* increased significantly, with *Bacillus cereus* showing the most pronounced growth. This suggests that *Bacillus cereus* possesses strong saline–alkali tolerance, which may be associated with plant adaptation to saline–alkali stress. Conversely, the populations of 23 endophytic bacterial species decreased, with *Alcaligenaceae*, *Aquabacterium*, *Paludibacter*, and *Rhizobium* exhibiting a significant decline, indicating their lower tolerance to saline–alkali conditions. In this experiment, it was discovered that *L. pumilum* exhibited a significant decrease in microbial species following saline–alkali stress. The number of *Bacillus cereus* was found to increase significantly after salt stress, demonstrating that plants can recruit *Bacillus cereus* to help overcome a salt environment.

The *Bacillus* species display remarkable resilience to a range of environmental conditions, including high temperatures, desiccation, radiation, acidity, salinity, and alkalinity [52]. They are capable of surviving for extended periods, even decades or thousands of years, in an adverse environment [53]. *Bacillus cereus* plays a role in promoting plant growth and enhancing plant tolerance to abiotic stresses [18,20]. *Bacillus cereus* is capable of producing antimicrobial substances, inhibiting the reproduction of harmful microorganisms, and enhancing the overall ecological environment [54]. Extraction of salt tolerance genes from the genomes of endophytic bacteria and their transfer into plants can enhance salt tolerance. The *acdS* gene from *Bacillus cereus* encodes ACC deaminase [55], which inhibits ethylene production. Therefore, Tian et al. introduced exogenous acdS genes into lily plants to enhance their tolerance to salt stress [56]. Genes related to nitrogen compound metabolism, phosphorus metabolism, potassium transport, IAA synthesis, iron binding, and antioxidant enzyme activities were identified in the LpBc-47 genome. These genes may play an important role in promoting plant growth and alleviating salt stress in LpBc-47.

The capacity to decompose cellulose is a significant indicator of probiotic bacteria [57]. LpBc-47 had the characteristics of cellulose decomposition (Figure 6d), which can facilitate the nutrient cycle of the soil and plant growth by decomposing plant cellulose and converting it into usable organic matter. Phosphorus is one of three essential elements for plant growth, yet most soils are deficient in this nutrient. The primary reason for this is that phosphorus is chemically fixed, and the capacity to dissolve the fixed phosphorus is regarded as a crucial indicator of the growth-promoting bacteria [58,59]. Nitrogen-fixing microorganisms are capable of facilitating plant growth and minimizing the utilization of nitrogen fertilizer [60]. LpBc-47 had the ability to dissolve phosphorus and fix nitrogen to increase the phosphorus and nitrogen in the soil (Figure 6e,f). In an oxidative environment, Fe^2+^ ions can be readily oxidized to insoluble Fe^3+^ ions. Endophytes are capable of specifically binding Fe^3+^ and reducing it to soluble iron [61]. Siderophore is a type of substance that has the specific ability to bind iron ions [62]. LpBc-47 had the ability to produce siderophore to specifically bind with iron ions (Figure 6h) and subsequently supply them with microorganisms, thereby facilitating their growth. Indoleacetic acid (IAA) is a growth hormone that assists plants in maintaining apical dominance, promoting seed germination, and facilitating fruit maturity [63]. LpBc-47 had the ability to produce IAA (Figure 6i) to promote plant growth. Plants produce significant quantities of ethylene in response to saline stress, which has an inhibitory effect on growth. Furthermore, ACC deaminase has the capacity to decompose the precursor substance of ethylene, 1-Aminocyclopropanecarboxylic acid, which consequently reduces the production of ethylene and thus protects the plants in question [64]. LpBc-47 had the ability to produce ACC deaminase (Figure 6j), which promoted plant growth in multiple ways.

The growth and salt tolerance (400 mM NaHCO_3_) of *L. pumilum* were investigated by watering the seedlings with LpBc-47. The experimental results indicated that the leaf length and dry weight of *L. pumilum* increased after the application of endophytic bacteria, suggesting that LpBc-47 promoted the growth of *L. pumilum*. Green fluorescent labeling protein (GFP) could precisely demonstrate the colonization site of the strain within the plant [65]. Through the transfer of GFP plasmid pHAP-II into LpBc-47, it was discovered that LpBc-47 colonized the roots and bulbs of *L. pumilum*, and the number of bacteria increased significantly under stress conditions. Malonic dialdehyde (MDA) is a significant product of membrane lipid peroxidation [66], and its production can exacerbate membrane damage and determine the degree of damage to the membrane system [67]. The main protective enzymes in the cell are superoxide dismutase (SOD), peroxidase (POD), and catalase (CAT) [68]. The primary function of SOD is to scavenge intracellular O_2_·^−^ in the plant cell, resulting in the generation of non-toxic O_2_ and less-toxic H_2_O_2_ [69]. CAT is effective in scavenging plant hydrogen peroxide in the body and catalyzing the decomposition of H_2_O_2_ [70]. POD operates surplus free radicals within the body, thereby enhancing plant stress tolerance [67]. During the process of plant growth, proline can assist proteins in binding with a greater quantity of water, thus preventing the dehydration and denaturation of proteins under conditions of osmotic stress [71]. Guo et al. observed that endophyte-treated Arabidopsis thaliana exhibited enhanced resistance to saline stress compared to wild plants [36]. The contents of MDA, O_2_·^−^, and H_2_O_2_ were significantly decreased, and the contents of POD, SOD, and CAT were significantly increased after the application of endophytic bacterial LpBc-47 treatments, indicating that endophytes can assist plants in removing detrimental ions and serve to scavenge excess free radicals in plants following the imposition of stress.

Microbial assistance offers several advantages over molecular improvement, including reduced research and development costs, rapid implementation, and the capacity to adapt to a diverse range of plants. As a typical plant growth-promoting bacterium, *Bacillus cereus* has been extensively studied in the agricultural field. *Bacillus cereus* can significantly enhance the growth and root activity of cucumber seedlings, while alleviating growth inhibition and photosynthetic suppression under saline–alkali stress by regulating antioxidant enzyme activities and improving photosynthesis [72]. The combination of *Bacillus cereus JYZ-SD2* with *Peniophora cinerea XC* enhances the salt tolerance of willow trees by increasing antioxidant enzyme activities, upregulating the expression of salt tolerance genes, and reducing Na absorption in roots, thereby mitigating salt stress-induced damage [73]. *Bacillus cereus G2* is capable of upregulating differentially expressed genes (DEGs) related to IAA signaling, ABA biosynthesis, signal transduction, and catabolism, as well as JA biosynthesis and signal transduction, which aids licorice by alleviating salt stress-induced damage [74]. Additionally, there are various strategies for utilizing *Bacillus cereus* as a microbial agent. Irrigating *Phaseolus vulgaris* with *Bacillus cereus CUAMS116* at different concentrations significantly promotes plant height and root proliferation [75]. When *Bacillus cereus* is used as a carrier-based biofertilizer (e.g., corn stover and coconut shell) for wheat, its survival rate increases without compromising its ability to promote plant growth and alleviate stress [49]. In this study, direct irrigation of *L. pumilum* with LpBc-47 markedly enhanced *L. pumilum* growth, increased leaf length and dry weight, and improved its salt–alkali tolerance under salt stress.

## 5. Conclusions

The microbial community composition of *L. pumilum* underwent changes under salt stress, leading to an increase in the abundance of beneficial microorganisms, which in turn helped to alleviate the adverse impacts of salt stress. The physiology and biochemistry of the isolated salt-tolerant endophytic bacterium LpBc-47 was investigated. The findings revealed that the organism was capable of growing under conditions of up to 400 mM NaHCO_3_ and exhibited optimal activity in a neutral environment. LpBc-47 possesses the probiotic characteristics of decomposing cellulose, dissolving phosphorus, fixing nitrogen, decomposing potassium, producing indole-3-acetic acid, synthesizing iron carriers, and decomposing ACC. The co-culture of LpBc-47 with plants can enhance plant growth and improve plant saline–alkali tolerance. Furthermore, LpBc-47 has been demonstrated to contribute to the resilience of salt-stressed seedlings. These results suggest that LpBc-47 is an effective biological agent for promoting plant growth and enhancing salt tolerance. 

## Figures and Tables

**Table 1:** Identification results of endophytic bacteria in *L. pumilum*.

Serial Number	Name
1	*Priestia aryabhattai*
2	*Pseudomonas putida*
3	*Actinomycetota bacterium*
4	*Psychrobacter pulmonis*
5	*Novosphingobium resinovorum*
6	*Cytophaga* sp.
7	*Sphingobium baderi*
8	*Flavobacterium*
9	*Stenotrophomonas geniculate*
10	*Cellvibrio diazotrophicus*
11	*Flavobacterium microcysteis*
12	*Sphingomonas* sp.
13	*Bacillus cereus*
14	*Ureibacillus chungkukjangi*
15	*Bacillus luti*
16	*Pseudomonas psychrotolerans*
17	*Bacterium*
18	*Serratia*
19	*Lysinibacillus sphaericus*
20	*Acinetobacter*
21	*Bacillus megaterium*
22	*Paenibacillus amylolyticus*
23	*Bacillus pumilus*
24	*Exiguobacterium*

**Table 2:** PGPR-related genes in LpBc-47 genome.

Gene Function	Genes Number	Genes Name
Nitrogen compound metabolic process	94	*Nif*, *nifD*
Phosphorus metabolic process	30	*PstA*, *pstB*, *pstC*
Potassium ion transport	88	*KdpC*, *KhtT*
Indole acetic acid synthase activity	6	*AdhR*, *aroA*
Antioxidant activity	10	*CAT*, *SOD*, *POD*
Iron ion binding	190	*EntS*, *dhbC*

## Data Availability

The original contributions presented in this study are included in the article. Further inquiries can be directed to the corresponding authors.

## Abbreviations

The following abbreviations are used in this manuscript:

LB	Lysogeny broth
PGPB	Plant growth-promoting bacteria
GFP	Green fluorescently labeled protein
NA	Nutrient agar
